# Advances in colon-targeted drug technologies

**DOI:** 10.1097/MOG.0000000000001064

**Published:** 2024-10-11

**Authors:** Charlotte Yeung, Laura E. McCoubrey, Abdul W. Basit

**Affiliations:** a University College London, School of Pharmacy, London; b Drug Product Development, GSK R&D, Ware, UK

**Keywords:** artificial intelligence and machine learning, colonic targeting, gastro-resistant film coatings, mesalamine formulations, oral drug delivery systems and the large intestine

## Abstract

**Purpose of review:**

Herein, we present an overview of innovative oral technologies utilized in colonic drug delivery systems that have made significant translational and clinical advancements to treat inflammatory bowel disease (IBD) in recent years.

**Recent findings:**

The colon is home to distinct physiological conditions, such as pH and microbiota, that have been exploited in the development of colonic drug delivery systems for the treatment of local and systemic diseases. However, given the intra and interindividual variability in the gastrointestinal tract of both healthy and diseased states, various systems have shown inconsistencies in targeted drug release to the colon. Recent breakthroughs have led to systems that incorporate multiple independent trigger mechanisms, ensuring drug release even if one mechanism fails due to physiological variability. Such advanced platforms have bolstered the development of oral biologics delivery, an especially promising direction given the lack of commercially available oral antibody medications for IBD. These concepts can be further enhanced by employing 3D printing which enables the personalisation of medicines.

**Summary:**

Leveraging these novel technologies can accurately deliver therapeutics to the colon, allowing for treatments beyond gastrointestinal tract diseases. To realize the full potential of colonic drug delivery, it is paramount that research focuses on the clinical translatability and scalability of novel concepts.

## INTRODUCTION

Colonic drug delivery has been an area of high interest over the past decades, with research being driven by improved therapeutic outcomes of local disorders such as inflammatory bowel disease (IBD), colorectal cancer and *Clostridioides difficile* infection (CDI), alongside systemic delivery of macromolecules such as protein and peptide drugs via oral routes [1^▪^,^2^,^3^]. A successful system requires a stable payload, which transverses the stomach and small intestine without premature release and degradation. There are physiological distinctions of the colon in comparison to the proximal gastrointestinal tract that can be leveraged, with key differences including pH and microbiota [3–6]. 

**Box 1:**
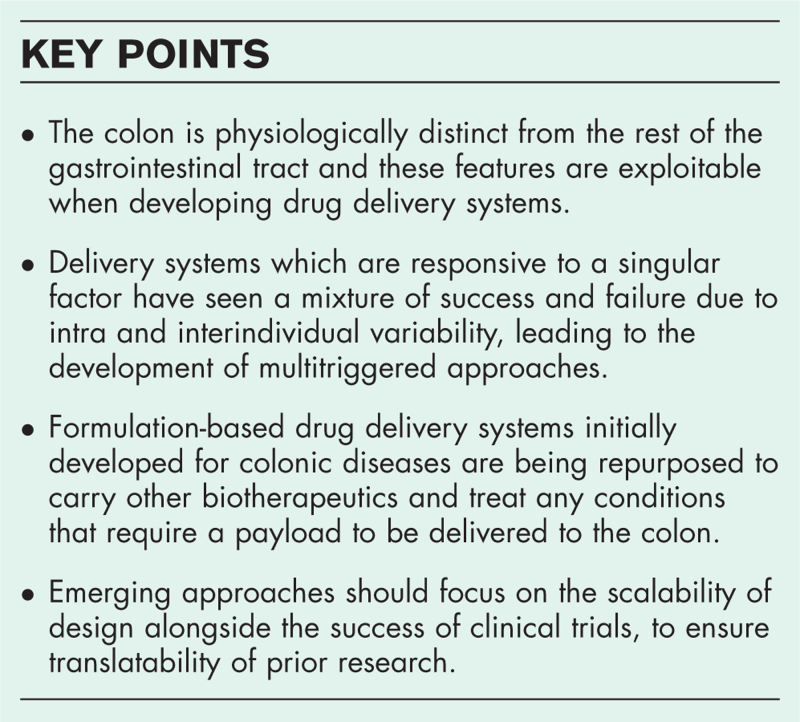
no caption available

**FIGURE 1:**
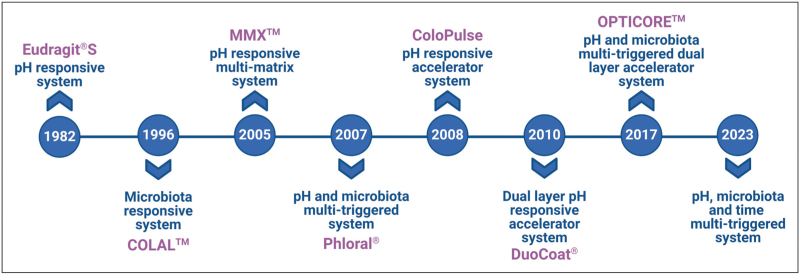
A chronological overview of colonic drug delivery platform advancements and the evolution of advanced technologies.

However, these characteristics are subject to great intra and interindividual variability, and gastrointestinal disorders can further alter colonic physiology [3,7]. It has been reported that colonic pH is reduced in patients with ulcerative colitis [8], IBD patients tend to have longer small intestinal transit times [9], and the gut microbiota and metabolic activity of those with IBD are significantly different compared to non-IBD control patients [10]. Similarly, other factors including diet, medication intake, and surgery affect the physiological factors typically associated with the colon [11,12]. A recent meta-analysis study has also highlighted substantial intra-subject variability in gastric content volume, which surpasses interindividual variability [13^▪▪^]. Additionally, the influence of sex was shown to be a factor, with females displaying lower volumes. The cumulative impact of these complexities has hindered the development of effective drug delivery systems. Consequently, innovative technologies have transitioned from approaches that rely on a single physiological factor for drug release to those which include a secondary, independent backup trigger, where drug release can occur in the presence of at least one of the two physiological factors, as seen in Fig. 1. This review explores the recent advances in colon-targeted drug technologies, ranging from products already on the market to the less common but promising approaches.

## pH-RESPONSIVE APPROACHES

The pH in the gastrointestinal tract varies according to biogeography and the first material that enabled formulations to utilize the gastrointestinal pH peak in the terminal ileum was Eudragit S (Evonik), a methacrylate copolymer used in enteric coatings, which dissolves when pH 7.0 is reached, facilitating drug release in the ileocolonic region [14]. Eudragit S has since been utilized in multiple marketed products to treat IBD, including Asacol and Lialda and for the delivery of mesalamine, and Budenofalk and Uceris for the delivery of budesonide. Lialdaand Uceris in particular utilize the Multi Matrix System (MMX) wherein a drug is incorporated into a hydrophilic and lipophilic matrix, and is enclosed by a pH-dependent polymer coating. The matrices allow for sustained drug release after the outer polymer has dissolved, to ensure a larger targeted area can be treated. However, reports have shown that Eudragit S alone can result in the premature or failed release of the drug due to the variability of pH in individuals and the lower pH often reported in IBD patients [3,15,16].

Further attempts have been made to enhance drug release in pH-responsive systems as seen with ColoPulse, a protective coating that combines Eudragit S and a disintegrant, such as croscarmellose sodium, in a single layer [17,18]. Once the pH threshold is reached in the gastrointestinal tract, fluid can penetrate the coating and reach the incorporated disintegrants which allows them to swell, rupture the polymer, and induce rapid disruption of the coating for accelerated drug release. The ColoPulse technology has been evaluated in the laboratory to deliver a variety of therapeutics and more recently has been used to deliver vitamins to explore microbiome-modulating capabilities, a study currently under evaluation in clinical trials [19,20]. Subsequent to the adoption of single-layer pH-dependent coatings, multilayer systems were introduced, including DuoCoat, a commercialized coating system made up of two layers. The inner layer is comprised of Eudragit S with a buffer salt which, when in contact with luminal fluid, triggers increased ionic strength and buffer capacity. This in turn accelerates the dissolution of the outer layer of pure Eudragit S leading to faster overall drug release [21]. Despite their potential, these platforms are constrained by the need for a minimum pH of 7 in the gastrointestinal tract, which has sparked the development of multifactor triggered approaches that will be discussed later in this review.

## MICROBIOTA-RESPONSIVE APPROACHES

Density of the microbiota and their resulting enzymatic reactions increases along the gastrointestinal tract, a feature that can be harnessed for microbiota-responsive colonic delivery systems [22].

Colon-targeted prodrugs have experienced clinical success in the forms of traditional azo prodrugs such as sulfasalazine, olsalazine, and balsalazide, which are activated through the cleavage of the azo-bond by the azoreductase enzymes produced by the colonic microbiota [23]. However, the design of prodrugs is drug-specific due to restrictions in chemical structure, alongside solubility and bioavailability requirements of the active drug [24]. In contrast, the formulation-based approach to drug design offers a more versatile solution whereby a single formulation can be applied to multiple drugs, providing a standardized release mechanism, as can be seen in the case of polysaccharides.

Certain polysaccharides such as pectin, chitosan, xanthan gum, and guar gum have been investigated in colonic delivery systems as they are metabolized by microbial enzymes in the colon to release their drug load [25]. Starch is another promising polysaccharide, specifically amorphous amylose, a retrograded starch, which has shown resistance against pancreatic enzymes but is digestible by colonic bacteria [26]. The combination of amylose with the water-insoluble polymer, ethylcellulose, is required to control the rate of swelling and ensure drug release occurs in the colon since the use of amylose alone leads to excessive swelling effects in the presence of water [27]. This concept (COLAL) has shown increased site-specific targeting in comparison to a pH-responsive system in healthy humans [28]. COLAL was further used to coat prednisolone metasulfobenzoate, producing a microbiota-responsive treatment for ulcerative colitis: COLAL-PRED that proceeded to Phase III clinical trials [29]. However, despite having significantly lower incidences of steroid-related adverse events, the formulation failed to show an equal or higher clinical efficacy over conventional prednisolone, thereby missing its primary endpoint. A variation of this technology using pectin as opposed to amylose gave rise to SmPill, used to deliver ciclosporin for the treatment of ulcerative colitis (CyCol) [30,31]. Whilst safety and tolerance levels were promising in an initial Phase II study [32], a secondary Phase II study was terminated for nonsufficient treatment remission in comparison to the placebo group [33].

In an attempt to bridge preclinical and clinical testing results, Ferraro *et al.*[34^▪^] looked to identify which polysaccharides can provide species-independent colon targeting. A systematic screening of seventeen different polysaccharide-coated mesalamine pellets was tested in faecal samples of IBD patients, IBD model rats, and healthy dogs. The combination of pellets coated with aloe vera extract:ethylcellulose and reishi extract: ethylcellulose showed the most promising results with similar release profiles across the three kinds of faecal samples. However, individual evaluation of every polysaccharide can be time-consuming and labour-intensive, often resulting in numerous unsuccessful outcomes. To address this issue, Abdalla *et al.*[35^▪^] successfully employed machine learning to predict mesalamine release from polysaccharide coatings in human, rat, and dog models in IBD-simulated colonic environments. This innovative approach can significantly streamline the selection of polysaccharides for laboratory evaluation, accelerating the development of novel drug delivery platforms.

A drawback of prodrug or polysaccharide-based systems is dysbiosis of the human microbiome that could cause required microbiota or enzymes to be absent. With disease states impacting dysbiosis, there is a need for therapeutic systems that are not constrained by a single element [36].

## MULTI-TRIGGERED RELEASE APPROACHES

As discussed earlier, the reliance on a single physiological factor often leads to failed delivery systems which either release drugs prematurely in the stomach/small intestine or their mechanism fails to activate due to the required trigger not being present in certain patients [3]. The variability in these factors across individuals has led to the development of systems that integrate multiple independent trigger mechanisms within a single layer (multitriggered release systems). This redundancy mitigates the risk of drug release failure since if one mechanism fails, the others can still trigger release, providing a fail-safe mechanism that enhances the reliability of drug delivery to the target site.

The first innovative multitrigger approach that has been commercialized is Phloral, a dual-trigger (pH and microbiota) coating for colonic drug delivery, composed of Eudragit S and resistant starch [37,38]. While Eudragit S dissolves at pH 7, should a patient's ileal pH be lower, then the resistant starch can instead be metabolized by the colonic microbiota to release the drug. In juxtaposition, if a patient does not have the required microbiota to metabolize the starch, as long as their ileal pH reaches 7, the coating will dissolve. Not only has Phloral shown effective results in IBD treatment [37], but the coating has also been utilized in clinical faecal microbiota transplantation (FMT) capsule studies to treat CDI [39], and clinical studies to treat obesity successfully [40]. Additionally, it has been used in two oral formulations for the treatment of Type II Diabetes: BioKier's butyrate (BKR-017) and glutamine (BKR-013) tablets [41].

**FIGURE 2:**
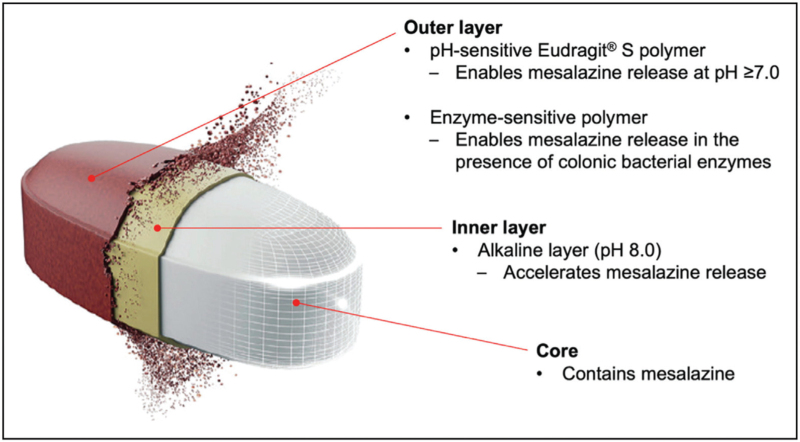
Layer-by-layer explanation of the OPTICORE coating technology used in Asacol 1600 mg mesalamine drug. Reused with permission from [3].

Further advancements in Phloral technology have led to a second marketed technology, OPTICORE, a pH and bacterial enzyme dual-triggered system, which uses a multilayer design [42,43]. The inner layer of OPTICORE is the same inner layer of DuoCoat, and the outer layer is Phoral. Mechanisms of accelerated drug release (mentioned above) are combined with independent triggers for drug release and can be visualized in Fig. 2. Since its release, OPTICORE has been used to deliver mesalamine to target colonic inflammation in IBD as the colon-targeting feature in Asacol 1600 mg (Octasa1600 mg). Having successfully passed Phase III clinical trials [44], Asacol 1600 mg is now commercially available worldwide, and surpasses the dosage of all previously approved oral mesalamine formulations.

A more recent enhancement to a dual-triggered approach is the combination of pH, microbiota, and a third element relating to intestinal transit time, giving rise to a triple-triggered oral delivery platform for colonic release with expectations to provide even greater precision [45^▪▪^]. In this system, the drug core is protected by an inner, swellable, time-dependant hydroxypropyl methylcellulose (HPMC) layer, and an outer coating made from a blend of pH-responsive Eudragit S, and microbially-degradable high-methoxyl pectin. The double-coated systems were tested with mesalamine *in vivo* on transgenic mice and benchmarked against commercial mesalamine, showing success in limiting the progression of inflammation and overgrowth of *Escherichia coli*. Promising results in these models encourage further studies of the system, perhaps in larger animal models before progression to clinical trials.

## DELIVERY OF BIOLOGICS

The colonic delivery of biologics to treat diseases including IBD and advanced colorectal cancer has been heavily researched. At present, all biologic antibody-related therapies are delivered via the parenteral route for IBD due to instability in the luminal environment and low permeability. However, there is a preference for orally administered therapeutics, shifting the interest to the delivery of biologics to treat IBD orally [2,46].

To address the issues of stability and permeability, Yadav *et al.*[47^▪^] have developed an antibody delivery platform composed of two features: a pH-sensitive coating, released only when exposed to specific pH and/or colonic bacteria, and a core consisting of amino acid-based excipients mixed with IL-6 antibodies (IL-6 is elevated in IBD patients) [48]. The excipients chosen stabilized the antibody against enzymes, allowing for an increased uptake via active and passive diffusion into the tissue. In-vivo results in DSS colitis mice showed increased tissue uptake of antibodies and downregulation of IL6 in comparison to conventional intraperitoneal injections, indicating its viability as an oral delivery system for monoclonal antibodies and potentially other biologics. Clinical steps have also been made with MB-001, an oral humanized antibody formulation for IBD that has entered clinical trials with the first patient dosed earlier this year [49]. The antibody is designed to have a targeted release of the drug in the inflamed mucosa, utilizing an oral sustained-release formulation.

To make further advancements in the delivery of biologics and other therapeutics, nano-based drug delivery systems (NDDS) have been heavily researched [50–52]. These systems have shown specific drug release at targeted inflammation sites via environmental triggers including pH, enzymes and rective oxygen species [53]. This further enhancement in disease specific targeting in IBD has shown promise in early stages [54]. However, the overarching issue of scalability of NDDSs of all therapeutics for marketed use remains due to their batch-to-batch variability and potential degradation [55].

## 3D-PRINTED DELIVERY SYSTEMS

One of the issues with conventional solid oral dosage forms is that, because they are manufactured at a large scale, there is no room for customizability to meet individual needs [56]. This is especially problematic because of the clinical variability across patients, reinforcing that a “one size fits all” approach may limit drug release efficacy in certain individuals. A potential solution to this comes in the form of 3D printing, which has recently propelled the field of personalized medicine through tailored dosage strengths and engineered release patterns and entered clinical studies [57–59].

In the field of colonic delivery, 3D printing has been applied to develop budesonide-targeted formulations [60,61^▪^]. In a recent in-vitro study, a pH-responsive, 3D printed colonic targeting budesonide tablet was developed, which showed sustained release in the colon, alongside targeting of specific areas such as the ileum and the proximal colon depending on the printed thickness level of the top outer layer [61^▪^]. The ability to control release allows for the treatment of IBD in different locations with varying doses.

The versatility of 3D printing is also shown in the encapsulation of liquid medicines, which has always proven to be a pharmaceutical challenge due to a lack of capsule formulations that can maintain stability through the gastrointestinal tract whilst carrying an aqueous product [62]. 3D printed capsules suitable to contain liquids were constructed for faecal transplants used to treat CDI, allowing for expedited screening of different designs and properties of the capsules, alongside rapid testing of a variety of formulations in comparison to conventional manufacturing approaches [63]. Other creative directions that have been taken include the printing of suppositories that have successfully carried tofacitinib citrate and budesonide, and infliximab [64,65], showcasing 3D printings’ success as a personalisable platform for IBD therapeutics. However, their scarcity in clinical use needs to be addressed before their viability can be fully assessed and compared to products on the market.

## CONCLUSION

The intra and interindividual variability in physiological parameters amongst both healthy and diseased states has hindered the development of effective colonic drug delivery systems. Issues with the reliability of systems responsive to a singular factor have since been addressed through the innovations of multitriggered delivery systems, using multiple independent trigger mechanisms to increase site-specific delivery and thus increase therapeutic effects against local diseases. These formulations originally designed for colonic diseases are now being adapted to deliver biological agents to treat a wider range of conditions outside of the gastrointestinal tract and can be personalized through the use of 3D printing technologies, ultimately expanding patient reach and improving their care.

## Acknowledgements

*None.*

### Financial support and sponsorship

*None.*

### Conflicts of interest

*Abdul W. Basit is co-inventor of the DuoCoat, Phloral and OPTICORE Technology and holds its patent [US20070243253A1].*

*The content of this commentary does not reflect the views of GSK.*

## REFERENCES AND RECOMMENDED READING

Papers of particular interest, published within the annual period of review, have been highlighted as:

▪ of special interest
▪▪ of outstanding interest
